# Persistent lymphopenia is a risk factor for ICU-acquired infections and for death in ICU patients with sustained hypotension at admission

**DOI:** 10.1186/s13613-017-0242-0

**Published:** 2017-03-17

**Authors:** Christophe Adrie, Maxime Lugosi, Romain Sonneville, Bertrand Souweine, Stéphane Ruckly, Jean-Charles Cartier, Maité Garrouste-Orgeas, Carole Schwebel, Jean-François Timsit, On behalf of the OUTCOMEREA study group, Jean-François Timsit, Elie Azoulay, Yves Cohen, Maïté Garrouste-Orgeas, Lilia Soufir, Jean-Ralph Zahar, Christophe Adrie, Michael Darmon, Corinne Alberti, Christophe Clec’h, Adrien Français, Aurélien Vesin, Stephane Ruckly, Frederik Lecorre, Didier Nakache, Aurélien Vannieuwenhuyze, Bernard Allaouchiche, Claire Ara-Somohano, Laurent Argault, Agnès Bonadona, Caroline Bornstain, Lila Bouadma, Alexandre Boyer, Christine Cheval, Jean-Pierre Colin, Anne-Sylvie Dumenil, Adrien Descorps-Declere, Jean-Philippe Fosse, Rebecca Hamidfar-Roy, Samir Jamali, Hatem Khallel, Christian Laplace, Alexandre Lautrette, Thierry Lazard, Eric Le Miere, Maxime Lugosi, Guillaume Marcotte, Laurent Montesino, Bruno Mourvillier, Benoît Misset, Delphine Moreau, Etienne Pigné, Stéphane Ruckly, Bertrand Souweine, Carole Schwebel, Gilles Troché, Marie Thuong, Guillaume Thierry, Dany Toledano, Eric Vantalon, Caroline Tournegros, Loïc Ferrand, Nadira Kaddour, Boris Berthe, Kaouttar Mellouk, Veronique Deiler, Kelly Tiercelet, Sophie Letrou, Igor Théodose, Julien Fournier

**Affiliations:** 1 Physiology Department, Cochin University Hospital, AP-HP, Paris Descartes University, 27 rue du Faubourg Saint Jacques, 75014 Paris, France; 2 Polyvalent ICU, Delafontaine Hospital, Saint-Denis, France; 3 Medical ICU, Grenoble 1 University, Albert Michallon Hospital, Grenoble, France; 4 Medical and Infectious Diseases ICU, Bichat University Hospital, AP-HP, Paris, France; 5 Clermont-Ferrand University, Medical ICU, Gabriel Montpied Hospital, Clermont-Ferrand, France; 6 UMR 1137 IAME Inserm- Paris Diderot University, 75018 Paris, France; 7 Polyvalent ICU, St Joseph Hospital, Paris, France

**Keywords:** Immunosuppression, Shock, ICU, Nosocomial, Infection, Survival, Absolute lymphocyte count

## Abstract

**Background:**

Severely ill patients might develop an alteration of their immune system called post-aggressive immunosuppression. We sought to assess the risk of ICU-acquired infection and of mortality according to the absolute lymphocyte count at ICU admission and its changes over 3 days.

**Methods:**

Adults in ICU for at least 3 days with a shock or persistent low blood pressure were extracted from a French ICU database and included. We evaluated the impact of the absolute lymphocyte count at baseline and its change at day 3 on the incidence of ICU-acquired infection and on the 28-day mortality rate. We categorized lymphocytes in 4 groups: above 1.5 × 10^3^ cells/µL; between 1 and 1.5 × 10^3^ cells/µL; between 0.5 and 1 × 10^3^ cells/µL; and below 0.5 × 10^3^ cells/µL.

**Results:**

A total of 753 patients were included. The median lymphocyte count was 0.8 × 10^3^ cells/µL [0.51–1.29]. A total of 174 (23%) patients developed infections; the 28-day mortality rate was 21% (161/753). Lymphopenia at admission was associated with ICU-acquired infection (p < 0.001) but not with 28-day mortality. Independently of baseline lymphocyte count, the absence of lymphocyte count increase at day 3 was associated with ICU-acquired infection (sub-distribution hazard ratio sHR: 1.37 [1.12–1.67], p = 0.002) and with 28-day mortality (sHR: 1.67 [1.37–2.03], p < 0.0001).

**Conclusion:**

Lymphopenia at ICU admission and its persistence at day 3 were associated with an increased risk of ICU-acquired infection, while only persisting lymphopenia predicted increased 28-day mortality. The lymphocyte count at ICU admission and at day 3 could be used as a simple and reproductive marker of post-aggressive immunosuppression.

**Electronic supplementary material:**

The online version of this article (doi:10.1186/s13613-017-0242-0) contains supplementary material, which is available to authorized users.

## Background

Lymphopenia is defined as a decrease below normal value (often 1.5 × 10^3^ cells/µL) of the blood circulating lymphocyte count; it reflects an impairment of the adaptive immune system. Several diseases can cause lymphopenia; they are associated with a higher risk of infection and adverse outcome [1, 2].

In critically ill patients, especially those with septic shock, after an initial phase of immune system hyperstimulation, dysfunction could appear secondarily. This is often called post-aggressive immunosuppression or compensatory anti-inflammatory response syndrome (CARS). It affects the innate and adaptive immune system [3, 4]. There is an increase in the level of anti-inflammatory cytokines, e.g., interleukin (IL)-10, in contrast to the decrease in pro-inflammatory cytokines levels, such as IL-6 or TNF-α. Immune cells are altered in both dimensions, qualitatively, and also quantitatively, as demonstrated with cells of innate immunity [5–7]. Persistence of CARS is associated with the risk of ICU-acquired infections and adverse outcome [7, 8].

Studies have shown the impact of critical illness on lymphocyte apoptosis and anergy [9–12]; however, there are few reports about the prognostic value in ICU of total lymphocyte count at admission and its evolution. These studies often evaluated the association between adverse outcome and other biomarkers of lymphocyte dysfunction than the lymphocyte count. However, the lymphocyte count would be a simple and reproducible marker of CARS. It was shown that low absolute lymphocyte counts are predictive of postoperative sepsis and a better predictor of bacteremia than conventional markers in patients admitted in emergency care units [13, 14]. Furthermore, a very recent study showed that persistent lymphopenia on the fourth day after bacteremia diagnosis predicts early and late mortality in those patients, including in the subgroup of patients with sepsis [15].

The main objective of this study was to evaluate the risk of development of an ICU-acquired infection according to the absolute lymphocyte blood count at admission and its evolution at day 3. The second objective was to evaluate how these parameters impact the 28-day mortality.

## Methods

We performed a retrospective study on data prospectively collected within the cohort study conducted with centers participating to the OUTCOMEREA database (OutcomeRea^®^).

### Ethical issues

This study was approved by our institutional review board (CECIC Clermont-Ferrand—IRB n°5891; Ref: 2007–2016), which waived the need for signed informed consent of the participants, in accordance with French legislation on non-interventional studies. However, the patients and their next of kin were asked whether they were willing to participate in the database, and none declined participation.

### Data collection

Data were prospectively collected daily by senior physicians in the participating ICUs. For each patient, the data were entered into electronic case report forms using VIGIREA^®^ and RHEA^®^ data capture software, and all case report forms were then entered into the OutcomeRea^®^ data warehouse. All codes and definitions were established prior to study initiation. For each patient, age, sex, and McCabe score were recorded. Severity of illness was evaluated on the first ICU day using the Simplified Acute Physiology Score (SAPS II), Sequential Organ Failure Assessment (SOFA) score, and Glasgow Coma Scale (GCS) score, and Knaus’ scale definitions were used to record preexisting chronic organ failures including respiratory, cardiac, hepatic, renal, and immune system failures. Admission category (medical, scheduled surgery, or unscheduled surgery), admission diagnosis (cardiac, respiratory, or neurological failure, infection, and other), invasive procedures (arterial or venous central catheter, Swan-Ganz catheter, or endotracheal intubation), and treatment of organ failures (inotropic support, hemodialysis, and mechanical ventilation) and the use of corticosteroids, gastro-protective drugs, and antibiotics were also recorded. Daily lymphocyte counts were retrospectively collected from four ICUs participating to OUTCOMEREA database between July 2006 and May 2012. All patients with a lymphocyte count in the first day of admission were included in the study. In order to avoid confusion bias, we excluded patients with chronic lymphocytic leukemia (CLL), infection with the human immunodeficiency virus (HIV) or aplasia at admission. We also excluded patients with limitation of life-sustaining therapy in the four first days after admission. Patients with shock or persistent low blood pressure below 90 mmHg of systolic blood pressure in the first day of admission were included. Study variables were the first lymphocyte count on the first day of admission and its evolution at day 3 after admission. The lymphocyte count at admission was categorized in four predefined classes: normal (>1.5 × 10^3^ cells/µL); subnormal (1 × 10^3^ cells/µL < lymphocytes ≤1.5 × 10^3^ cells/µL); low (0.5 × 10^3^ cells/µL < lymphocytes ≤1 × 10^3^ cells/µL); very low (≤0.5 × 10^3^ cells/µL).

The evolution of lymphocyte count at day 3 versus baseline was defined as a binary variable: normal count (≥1.5 × 10^3^ cells/µL) or relevant increase (more than 0.2 × 10^3^ cells/µL) and decrease or no relevant increase (≤0.2 × 10^3^ cells/µL). We handled missing values at day 3 (n = 166, 22.1%) by taking the value one day before or after.

Nosocomial infection was defined as bacteremia, pneumonia, or catheter-related infection occurring after 72 h from admission. Definition of nosocomial infection provided from the HELICS (Hospital in Europe Link for Infection Control through Surveillance) project [16]. Bacteraemia was defined as the presence of pathogenic bacteria in blood culture. Pneumonia was defined as a chest X-ray with suggestive image of pneumonia with clinical and biological signs of pulmonary infection associated with a positive quantitative bacteriological culture from a respiratory sample: a broncho-alveolar lavage [BAL ≥10^4^ colony-forming unit (CFU)/ml]; a protected specimen brush (≥10^3^ CFU/ml); a blind protected bronchial sampling (≥10^3^ CFU/ml); a tracheal aspiration (≥10^5^ CFU/ml). Catheter infection was defined as positive quantitative catheter culture (≥10^3^ CFU/ml) treated by physicians in charge. Only the first event was considered for analysis.

### Statistical analysis

Characteristics of patients were described as count (percent) or median [interquartile range, IQR] for qualitative and quantitative variables, respectively, and were compared between groups using Chi-square or Mann–Whitney tests, as appropriate.

In order to decrease the risk of confusion bias between lymphopenia and acquired-ICU infection, we developed a propensity score aimed to predict the probability to have a nosocomial infection conditionally on variables recorded in the first 2 days of admission [17].

A logistic regression was used to construct the propensity score including variables on clinical relevance or statistic comparison on univariate analysis. Linearity of the logit of continuous covariates was checked. The following clinically relevant variables were entered in the model: age, gender, admission category, center, Knaus definitions, McCabe score, main reason for ICU admission (multi-organ failure, cardiogenic shock, septic shock, coma, acute respiratory deficiency), diabetes with complications (binary variable), severity illness related to specific organ assessed by the sequential SOFA score categorized in 2 classes, lower or equal to two or higher (cardiovascular, neurological, hepatic, renal, coagulation failures), acute respiratory distress syndrome, mechanical ventilation, central venous catheter, arterial catheter or arterial pulmonary catheter, temperature, use of gastro-protective drugs, antibiotics, or corticosteroids.

Then, an inverse probability of treatment weighted (IPTW) [18] based on the propensity score was computed to create a pseudo-population in which the probability to develop or not an ICU-acquired infection was equal. We performed a model with covariates using for the construction of the propensity score weighted by the IPTW including the explicative variables, baseline lymphocyte count, and evolution at the third day [19]. We took the 5–95th percentiles of IPTW to create a new pseudo-population to assess the robustness of the model.

Sub-distribution hazard ratios (sHRs) were developed to assess the independent effects of lymphocyte count at admission and the evolution at day 3 on subsequent risk of ICU-acquired infection. Discharge alive from ICU was treated as competing events. Data were censored at 28 days since the fourth day after admission.

For the secondary objective, risk of death related to initial lymphocyte count and its evolution at day 3, the same protocol was used. We developed a specific propensity score aiming to predict the probability to die in ICU within 28 days of inclusion conditionally on variables recorded within the first 2 days of admission. The following clinically relevant variables were entered in the model: age, gender, admission category, center, Knaus definitions, cardiogenic shock as symptom at admission, continuous monitoring as reason of admission, complicated diabetes, severity illness related to specific organ assessed by the SOFA score categorized in two classes, lower, or equal to 2 or higher (cardiovascular, neurological, hepatic, renal, coagulation failure), respiratory failure severity reflected by acute respiratory distress syndrome, requiring invasive mechanical ventilation, central venous catheter, arterial catheter or arterial pulmonary catheter, temperature, use of corticosteroids.

Sub-distribution hazard ratios (sHRs) were developed with covariates using for the construction of the propensity score weighted by the IPTW. Discharge alive from ICU was treated as competing events. For all analyses, p < .05 was considered to statistically significant. All analyses were performed using SAS, version 9.3 (SAS Institute, Cary, NC, USA).

## Results

### Population description

**Fig. 1:**
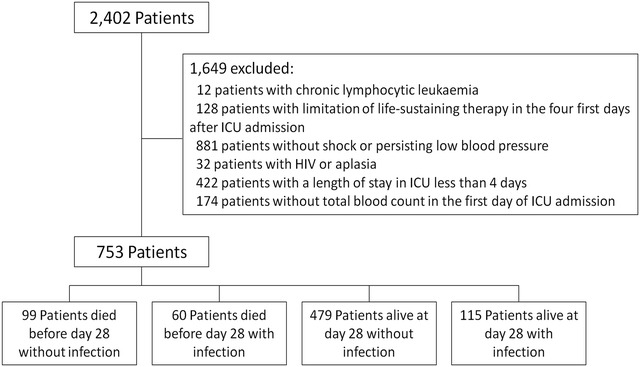
Flowchart

Of the 2402 patients recorded within the 4 participating ICUs (Fig. 1), 753 patients were included. The mean age was 68 [56; 78] years, 467 patients (62%) were males, and the median SOFA score at admission was 8 [5, 11]. Medical admission represented the most frequent cases [596 patients (79%)], and septic shock was the first diagnosis at admission in 154 patients (21%). Mechanical ventilation was required for 559 patients (74%) and vasoactive agents at day 1 or 2 for 480 patients (63.8%). The median length of stay in ICU was 9 days [6–18]. A total of 174 (23%) patients had ICU-acquired infection and 161 (21%) patients died in ICU during the study period (Table 1).

**Table 1:** Patients’ characteristics at admission

Variable	Population N = 753	No ICU-acquired infection (N = 579)	With ICU-acquired infection (N = 174)	P value	Alive (N = 592)	Dead (N = 161)	P value
Age	68 [56–78]	67.6 [56–78]	69 [55–77]	0.4106	66.5 [55–77]	71.5 [59–79]	0.02
Men	467 (62)	342 (59)	125 (72)	0.0023	359 (61)	108 (67)	0.13
Length of stay (days)	9 [6–18]	7 [5–13]	23 [14–37]	<.0001	9 [5–19]	10 [7–17]	0.18
Center							
A	501 (66.5)	402 (69)	99 (57)	0.0030	406 (69)	95 (59.0)	0.002
B	105 (14)	80 (14)	25 (14)		86 (14)	19 (12)	
C	35 (4.6)	21 (3.6)	14 (8.0)		27 (4.6)	8 (5.0)	
D	112 (15)	76 (13)	36 (21)		73 (12)	39 (24)	
Admission category				0.7400			0.003
Medical	596 (79)	457 (19)	139 (80)		454 (77)	142 (88)	
Unscheduled surgery	104 (14)	79 (14)	25 (14)		94 (16)	10 (6)	
Scheduled surgery	53 (7)	43 (7)	10 (6)		44 (7)	9 (6)	
Co-morbidities (Knaus definitions)							
Chronic hepatic failure	45 (6.0)	41 (7)	4 (2.3)	0.0196	30 (5)	15 (9)	0.044
Chronic cardiovascular failure	101 (13.4)	70 (12)	31 (18)	0.0519	70 (12)	31 (19)	0.014
Chronic respiratory failure	157 (20.8)	120 (21)	37 (21)	0.8780	126 (21)	31 (19)	0.57
Chronic renal failure	61 (8.1)	47 (8.1)	14 (8.0)	0.9758	44 (7.4)	17 (10.6)	0.19
Immunosuppression	69 (9.2)	54 (9.3)	15 (8.6)	0.7772	59 (10.0)	10 (6.2)	0.14
Long-term corticosteroids use	24 (3.2)	19 (3.3)	5 (2.9)	0.7882	20 (3.4)	4 (2.5)	0.57
History of chemotherapy	40 (5.3)	31 (5)	9 (5.2)	0.9254	31 (5)	9 (5)	0.86
Main reason of admission							
Coma	106 (14)	81 (14)	25 (14)	0.8999	81 (14)	25 (15)	0.55
Acute respiratory failure	211 (28.0)	150 (26)	61 (35)	0.0184	164 (27.7)	47 (29)	0.71
Septic shock	154 (20.4)	123 (21)	31 (18)	0.3257	121 (20)	33 (20)	0.99
Cardiogenic shock	39 (5)	14 (4)	15 (8)	0.0195	22 (4)	17 (11)	0.0005
Hemorrhage shock	50 (6.6)	40 (7)	10 (6)	0.5895	43 (7)	7 (4)	0.19
Multi-organ failure	21 (3)	11 (2)	10 (6)	0.0069	14 (2)	7 (4)	0.17
Shock (other)	27 (3.6)	23 (4)	4 (2)	0.2978	21 (3.5)	6 (4)	0.91
Other	145 (19)	127 (22)	18 (10)	0.0007	126 (21)	19 (12)	0.007
SAPS II score	49 [37–60]	48 [3–59]	51 [40–62]	0.0374	47 [36–57]	57 [46–66]	<0.0001
SOFA score	8 [5–11]	8 [5–11]	10 [7–12]	<.0001	7.5 [5–11]	10 [7–12]	<0.0001
Cardiovascular SOFA score (>2)	462 (61)	333 (57)	129 (74)	<.0001	333 (56)	129 (80)	<0.0001
Mechanical ventilation	559 (74)	411 (71)	148 (85)	0.0002	422 (71)	137 (85)	0.0004
Antibiotic day 1 or 2	581 (77)	448 (77)	133 (76)	0.80	455 (78)	126 (78)	0.71

Data are expressed as number (%) or median [interquartile]. ICU: intensive care unit; SAPS II: Simplified Acute Physiology Score; SOFA: Sequential Organ Failure Assessment. Of note, in some cases, septic shock was not the cause of admission in ICU, but developed within the first hours of ICU admission

The median number of lymphocyte counts measurements was 6 [4–13]. The percentage of day with a lymphocyte count by patient during ICU stay was 75%, and the median range between two blood samples with lymphocyte count was 1 day. The median of the lymphocyte count at admission was 0.80 [0.51–1.29] × 10^3^ cells/µL. The distribution in 4 classes was as follows: 149 patients (20%) had a normal lymphocyte count with a median of 1.97 [1.70–2.80] × 10^3^ cells/µL; 141 patients (19%) had a lymphocyte count ranging between 1 and 1.5 × 10^3^ cells/µL with a median of 1.19 [1.10–1.30] × 10^3^ cells/µL; 278 patients (37%) had a lymphocyte count ranging between 0.5 and 1 × 10^3^ cells/µL with a median of 0.72 [0.61–0.84] × 10^3^ cells/µL; 185 patients (24%) had a lymphocyte count lower than 0.5 × 10^3^ cells/µL with a median of 0.34 [0.24–0.43] × 10^3^ cells/µL.

Among the total of 174 (24%) ICU-acquired infections, pneumonia was diagnosed in 113 (64.9%) patients, bacteremia in 37 (21.3%) and catheter-associated infection in 36 (20.7%). In 13 patients, 2 sites of infection were diagnosed the same day. *Enterobacteriaceae* bacteria were the most frequent pathogens isolated, followed by *Pseudomonas* spp. and *Staphylococcus aureus* (Tables 2, 3).

**Table 2:** Description of ICU-acquired infection related to site of infection and time to event

	No (%)	Time to event (median [IQ]) or days of event
Total	174	8
Pneumonia	113 (64.9)	10 [6–15]
Bacteremia	37 (21.3)	8 [6–13]
Catheter-associated infection	36 (20.7)	8 [5–13]
Pneumonia with bacteremia	6 (3.4)	11.5 [7–23]
Catheter infection with bacteremia	3 (1.7)	13 [7–22]
Pneumonia with catheter-associated infection	3 (1.7)	13 [4–14]

Data are expressed as number (%) or median [interquartile]

**Table 3:** Description of ICU-acquired infection related to site of infection and microorganism (percentage of the total of pathogens isolated in a site)

Pathogens	Pneumonia (n = 113)	Bacteremia (n = 37)	Catheter infection (n = 36)
*Staphylococcus aureus*	21 (18.6)	6 (16.2)	3 (8.3)
Coagulase-negative Staphylococci	8 (7.1)	5 (13.5)	9 (25.0)
Other GPB	16 (14.2)	9 (24.3)	8 (22.2)
Fermenting GNP	46 (40.7)	13 (35.1)	14 (38.9)
Non-fermenting GNP	40 (35.4)	6 (16.2)	7 (19.4)
Anaerobes	1 (0.9)	1 (2.7)	0
Fungi	5 (4.4)	5 (13.5)	1 (2.8)
Polymicrobial	21 (18.6)	8 (21.6)	5 (13.9)
MDR pathogens	47 (45.6)	10 (27.0)	9 (25.0)

Data are expressed as number (%) or median [interquartile]. MDR: multi-drug-resistant, including methicillin-resistant *Staphylococcus* *aureus*, *Enterobacteriaceae* resistant to third-generation cephalosporins, *Pseudomonas aeruginosa* resistant to ticarcillin and/or imipenem and/or ceftazidime, *Stenotrophomonas maltophilia, Burkholderia cepacia*, and *Acinetobacter baumannii*. GPB; Gram-positive bacteria, GNB; Gram-negative Bacteria; non-fermenting GNB (*Pseudomonas* spp., *Acinetobacter baumannii, Stenotrophomonas maltophilia, Burkholderia cepacia*)

There were no relationships between the lymphocyte count and the SOFA score, and between the delta of the SOFA score and the variations in the lymphocyte counts. This result is consistent with our results about the independent role of immune paralysis and organ failures.

### Risk of ICU-acquired infection

**Fig. 2:**
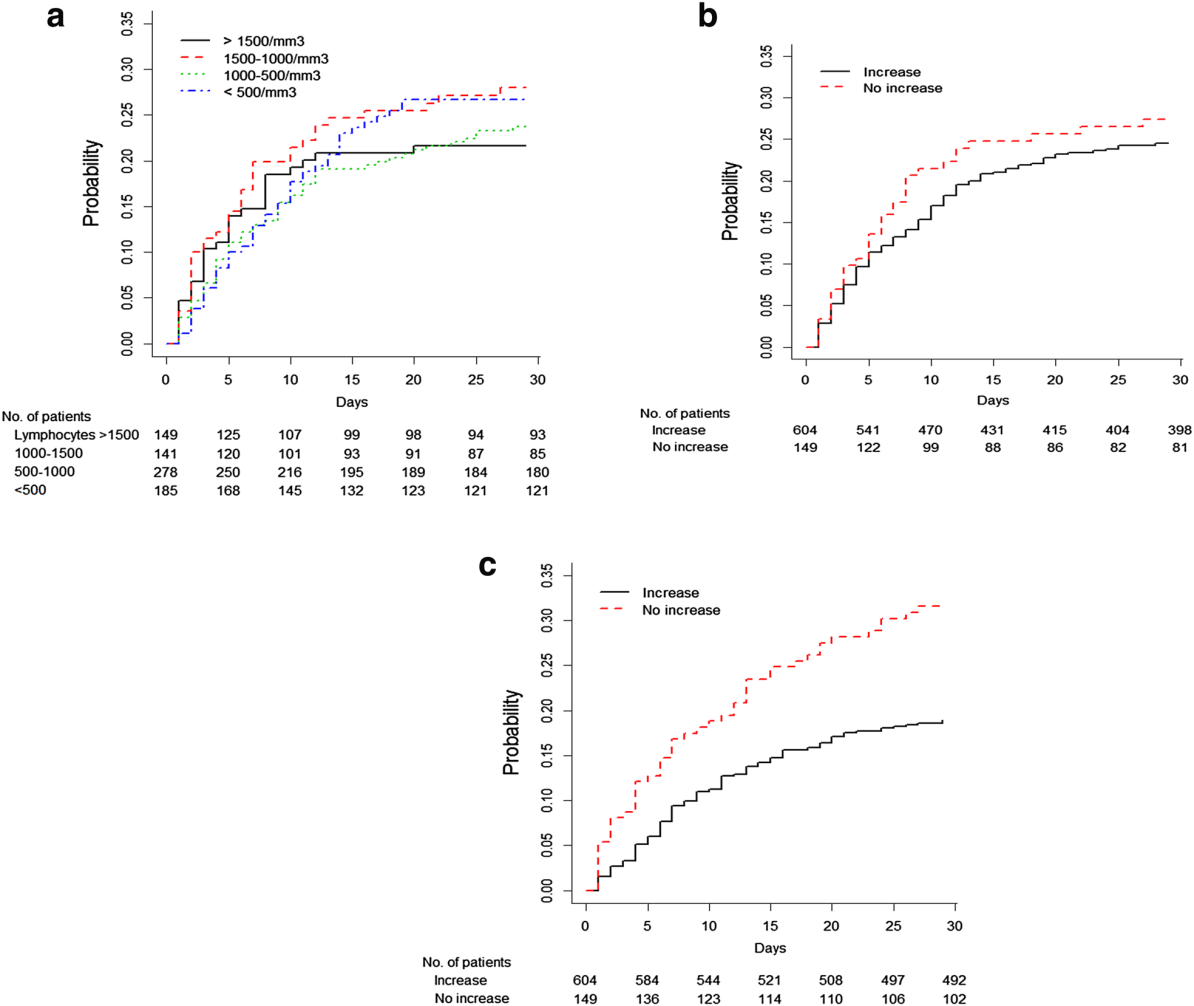
Cumulative incidence curves of ICU-acquired infection **a** according to baseline lymphocyte count categorized in 4 classes; cumulative incidence curve of ICU-acquired infection (**b**) and incidence curve of death (**c**) according to the increase from baseline of the lymphocyte count at day 3 (increase in lymphocyte count was considered significant if greater than 0.2 × 10^3^ cells/µL). Numbers below each figure represent the number of patients still at risk of event at a particular time point. No patient were lost to follow-up at day 28

Comparisons between patients with ICU-acquired infection and the others are shown in Table 1. The final logistic model used to calculate propensity score is given in Additional file 1: Table E1. The cumulative incidence curve of ICU-acquired infection is shown in Fig. 2a.

Sub-distribution hazard ratios (sHRs) of ICU-acquired infection were significant for abnormal values at admission (Table 4), with no difference between subnormal and very low lymphocyte counts. The absence of relevant increase in the lymphocyte count at day 3 was associated with an increased risk of developing an infection (sHR of 1.37 [1.12–1.67], *p* = 0.002) (Fig. 2b). The interaction term between baseline lymphocyte count and lymphocyte increase at day 3 was not significant. Importantly, the onset of ICU-acquired infection was associated with an increased day-28 mortality (p < 0.001).

**Table 4:** Results of the sub-distribution Hazard ratio (sHR) of baseline lymphocyte count and its evolution at day 3 for the risk of ICU-acquired infection (adjusted with the covariates used in the propensity score of acquiring a nosocomial infection before day 28 using an IPTW estimator; see Additional file 2)

Variables	sHR	IC-95		p value
Baseline lymphocyte count categorized in 4 classes				0.001
Normal value ≥1.5 × 10^3^ cells/µL	Reference	–	–	
Subnormal class (<1.5 and ≥ 1 × 10^3^ cells/µL)	1.60	1.24	2.08	0.0004
Low class (<1 × 10^3^ cells/µL and ≥0.5 × 10^3^ cells/µL)	1.43	1.12	1.85	0.004
Very low class (<0.5 × 10^3^ cells/µL)	1.63	1.23	2.15	0.0006
Non-significant increase (below 0.2 × 10^3^ cells/µL) at day 3 and abnormal value	1.37	1.12	1.67	0.002

### Risk of 28-day mortality

Comparisons between patients’ dead in ICU and others are shown in Table 1, using the final logistic model used to calculate propensity score (Additional file 1: Table E2). The incidences of 28-day mortality according to baseline lymphocyte count and its evolution at day 3 are shown in Table 5. The baseline count of lymphocyte had no impact on the 28-day mortality in ICU. However, the decrease or the non-significant increase on day 3 was significantly associated with the death in ICU [sHR of 1.67 [1.37–2.03], p < 0.0001 (Table 5)]. The cumulative incidence curve of death according to the evolution of lymphocyte count is represented in Fig. 2c.

**Table 5:** Results of the sub-distribution Hazard ratio (sHR) of baseline lymphocyte count and its evolution at day 3 for the risk of 28-day ICU mortality (adjusted with the covariates used in the propensity score of dying before day 28 using an IPTW estimator; see Additional file 2)

Variables	sHR	IC-95		p value
Baseline lymphocyte count categorized in 4 classes				0.15
Normal value ≥1.5 × 10^3^ cells/µL	Reference	–	–	
Subnormal class (<1.5 and ≥ 1 × 10^3^ cells/µL)	0.84	0.658	1.08	0.176
Low class (<1 × 10^3^ cells/µL and ≥0.5 × 10^3^ cells/µL)	1.09	0.891	1.36	0.377
Very low class (<0.5 × 10^3^ cells/µL)	0.99	0.773	1.28	0.969
Non-significant increase (below 0.2 × 10^3^ cells/µL) at day 3 and abnormal value	1.67	1.37	2.03	<0.0001

## Discussion

To our knowledge, our study is the first large cohort study which evaluated the relation between the baseline lymphocyte count and its evolution at day 3, and the risk of ICU-acquired infection and death in patients admitted in ICU with sustained hypotension. We demonstrated the significant independent prognostic impact of a low lymphocyte count at baseline on the risk to develop an ICU-acquired infection. A persisting lymphopenia or a non-significant increase at day 3 is associated with a risk to develop a nosocomial infection and with increased 28-day mortality (Additional file 1).

Acute critical ill patients, particularly in case of sepsis, often present signs of systemic inflammatory response syndrome (SIRS) which could be related to pro-inflammatory response. Beside this pro-inflammatory response, an anti-inflammatory response occurs. In these patients, several studies showed increased secretion of anti-inflammatory cytokines, e.g., IL-10, and decreased activation of immunity cells, e.g., monocytes [20, 21]. Thus, the immune response can display various profiles: combined anti- and pro-inflammatory response; anti-inflammatory response; or global immune depression. This syndrome of acquired deficiency of immune system is called the post-aggressive immunosuppression or compensatory anti-inflammatory response syndrome (CARS) [3, 4]. This secondarily impaired immunity has been described for decades [9]; several studies correlated it with poor outcome [5–7, 22]. This could explain the onset of nosocomial infections with opportunistic microorganism in septic patients, e.g., viral reactivation or fungal infection [23–25].

CARS involves both the innate and adaptive parts of immune system. It affects different cells involved in the innate immune system, such as polymorphonuclear neutrophils, dendritic cells, and monocytes. The link with a poor outcome was demonstrated in several studies [4, 26]. Monocytes dysfunction is now evaluated by a clinically validated surrogate marker: mHLA-DR expression [4, 27]. While biological testing of mHLA-DR expression is standardized [10] and then could offer a well-recognized biological test to select patients who would benefit of immune-adjuvant therapy, this test is not yet generalized in clinical practice.

The acquired immunity cells such as lymphocytes are also affected. Lymphocytes, particularly T-cells subset, are a cornerstone of the adaptive response to aggressions. An acquired or congenital lymphocyte deficiency increases the risk of infection and of death. CARS is correlated with lymphocyte function alteration, which has been described for 30 years. Function alteration is reflected by a decreased production of pro-inflammatory cytokines, such as IL-2, an increased production of anti-inflammatory cytokines, such as IL-10, an increased expression on cells membrane of inhibitory receptor such as programmed cell death-1, and a decreased expression of T-cell receptor repertoire diversity [27–31]. While our understanding of the mechanism of lymphocyte alteration during sepsis progresses, the link with patient’s prognosis is not always established.

An increased apoptosis was described in patients [12, 22]. Various pathways seem to be involved in the lymphocyte apoptosis in case of sepsis: an extrinsic pathway, mediated by the caspase-8, and an intrinsic pathway, mediated by the caspase-9 [10, 22]. In the study of Le Tulzo et al. [22], the magnitude of apoptosis was correlated with the persistence of multi-organ dysfunctions, duration of mechanical ventilation, and death. The correlation between the quantitative alteration of lymphocyte and a poor outcome was shown in two studies involving children [12, 32, 33]. In 21 adult patients with septic shock, Venet et al. [12] also described a median lymphocyte count within the first 24 h following admission for septic shock close to our results (0.5–0.7 × 10^3^ cells/µL). Altered lymphocyte function with recombinant human IL-7 or anti-programmed cell death-1 antibody may be promising targets for future clinical studies [27].

In a retrospective study of bacteremic patients, an association was observed between persistent lymphopenia (defined as below of 0.6 × 10^3^ cells/µLon the fourth day) with the 28-day mortality (primary endpoint), 1-year mortality, and subsequent hospital infection [15]. However, the low baseline total lymphocyte count (≤0.6 × 10^3^ cells/µL) was not associated with any of them, conversely to what we observed in our study. This difference may be due to the lymphopenia threshold definitions, and also to the case mix, as we included all patients with sustained hypotension, whether or not they had sepsis and/or bacteremia. As a matter of fact, we included all patients with an unstable hemodynamic status, in order to take into account the severity of patient as a promoter of CARS. Indeed, dysfunction of immune system was observed not only in septic patients, but also in post-traumatic or severely burned patients [34–37].

Although our study did not provide information on the link between the lymphocyte count and the qualitative alteration of lymphocyte function, it is the first one that demonstrated in a large cohort of patients, the impact of a low lymphocyte count at ICU admission and of its persistence on the risk to develop an ICU-acquired infection and of increased mortality. The interaction between the lymphocyte count at baseline and its evolution found in our study could reflect the persistent status of post-aggressive immunosuppression. Of course, our study did not preclude the absence of added prognostic value of the lymphocyte subsets, which has already been reported in the literature [22, 28, 38]; however, it highlights that the routinely measured total lymphocyte count may be taken into account. Indeed, the total lymphocyte count is simple to evaluate, without any special skill or laboratory equipment. However, further studies are warranted to figure out whether or not functional new markers would add more information that plain absolute lymphocyte counts.

Case mix varied between centers, which may explain significant differences between numbers of exams performed and mean lymphocyte counts between centers. However, we did not unmask heterogeneity between prognostic impacts of lymphocyte alterations between centers. We cannot make any causative relationship between mortality and ICU-acquired infection with low lymphocyte count, as they may all be related to the disease severity. Also, we do have any data on the immunoresponse and the anergy–apoptosis of this lymphocyte in general and lymphopenia in particular, as it could be expected from a retrospective study that requires a prospective confirmation using the functional activities of the different lymphocytes involved in the inflammation processes.

### Conclusion

A large cohort of ICU patients with shock at admission, we demonstrated the independent impact of a low baseline lymphocyte count and its non-relevant increase at day 3 with the risk of ICU-acquired infection and, for persistent lymphopenia, its impact on 28-day mortality. Total lymphocyte count appears as a simple and routine marker of immune dysfunction, and might be useful for selecting patients that could benefit of potential immune-adjuvant therapies [27].

## Appendix: Members of the OUTCOMEREA study group

### Scientific committee

Jean-François Timsit (Hôpital Bichat, Paris and INSERM U823, Grenoble, France), Elie Azoulay (Medical ICU, Hôpital Saint Louis, Paris, France), Yves Cohen (ICU, Hôpital Avicenne, Bobigny, France), Maïté Garrouste-Orgeas (ICU Hôpital Saint-Joseph, Paris, France), Lilia Soufir (ICU, Hôpital Saint-Joseph, Paris, France), Jean-Ralph Zahar (Microbiology Department, Hôpital Necker, Paris, France), Christophe Adrie (ICU, Hôpital Cochin, Paris, France), Michael Darmon (Medical ICU, University hospital St Etienne, France), Corinne Alberti (Robert Debré Hospital, Paris France) and Christophe Clec’h (ICU, Hôpital Avicenne, Bobigny, and INSERM U823, Grenoble, France).

### Biostatistical and informatics expertise

Jean-Francois Timsit (Hôpital Albert Michallon and Integrated Research Center U823, Grenoble, France), Corinne Alberti (Medical Computer Sciences and Biostatistics Department, Robert Debré, Paris, France), Adrien Français (Integrated Research Center U823, Grenoble, France), Aurélien Vesin (Integrated Research Center U823, Grenoble, France), Stephane Ruckly INSERM U823, Grenoble, France), Christophe Clec’h (ICU, Hôpital Avicenne, Bobigny, and INSERM U823, Grenoble, France), Frederik Lecorre (Supelec, France), and Didier Nakache (Conservatoire National des Arts et Métiers, Paris, France), Aurélien Vannieuwenhuyze (Tourcoing, France).

### Investigators of the Outcomerea database

Christophe Adrie (ICU, Hôpital Delafontaine, Saint Denis, France and Physiology, Hôpital Cochin, Paris, France), Bernard Allaouchiche (Surgical ICU, Edouard Herriot Hôpital, Lyon), Claire Ara-Somohano (CHU A Michallon, Grenoble, France), Laurent Argault (medical ICU Edouard Herriot Hôpital, Lyon), Agnès Bonadona (CHU A Michallon, Grenoble, France), Caroline Bornstain (ICU, Hôpital de Montfermeil, France), Lila Bouadma (ICU, hôpital Bichat Claude-Bernard, Paris, France), Alexandre Boyer (ICU, Hôpital Pellegrin, Bordeaux, France), Christine Cheval (SICU, Hôpital Saint-Joseph, Paris, France), Jean-Pierre Colin (ICU, Hôpital de Dourdan, Dourdan, France), Michael Darmon (ICU, CHU Saint Etienne), Anne-Sylvie Dumenil (Hôpital Antoine Béclère, Clamart France), Adrien Descorps-Declere (Hôpital Antoine Béclère, Clamart France), Jean-Philippe Fosse (ICU, Hôpital Avicenne, Bobigny, France), Rebecca Hamidfar-Roy (CHU A Michallon, Grenoble, France), Samir Jamali (ICU, Hôpital de Dourdan, Dourdan, France), Hatem Khallel (ICU, Cayenne General Hospital), Christian Laplace (ICU, Hôpital Kremlin-Bicêtre, Bicêtre, France), Alexandre Lautrette (ICU, CHU G Montpied, Clermont-Ferrand), Thierry Lazard (ICU, Hôpital de la Croix Saint-Simon, Paris, France), Eric Le Miere (ICU, Hôpital Louis Mourier, Colombes, France), Maxime Lugosi (CHU A Michallon, Grenoble, France), Guillaume Marcotte (surgical ICU, Edouard Herriot Hôpital, Lyon), Laurent Montesino (ICU, Hôpital Bichat, Paris, France), Bruno Mourvillier (ICU, Hôpital Bichat, France), Benoît Misset (ICU, Hôpital Saint-Joseph, Paris, France), Delphine Moreau (ICU, Hôpital Saint-Louis, Paris, France), Etienne Pigné (ICU, Hôpital Louis Mourier, Colombes, France), Stéphane Ruckly (Integrated Research Center U823, Grenoble, France), Bertrand Souweine (ICU, CHU G Montpied, Clermont-Ferrand), Carole Schwebel (CHU A Michallon, Grenoble, France), Gilles Troché (Hôpital Antoine, Béclère, Clamart France), Marie Thuong (ICU, Pontoise, France), Guillaume Thierry (ICU, Hôpital Saint-Louis, Paris, France), Dany Toledano (CH Gonnesse, France), and EricVantalon (SICU, Hôpital Saint-Joseph, Paris, France).

### Study monitors

Caroline Tournegros, Loïc Ferrand, Nadira Kaddour, Boris Berthe, Kaouttar Mellouk, Veronique Deiler, Kelly Tiercelet, Sophie Letrou, Igor Théodose, and Julien Fournier.

## Additional files

**Additional file 1.** Lymphocyte_data-set.

**Additional file 2.** Details on the model fitting propensity score used.

## Abbreviations

sHR: sub-distribution hazard ratio
ICU: intensive care unit
CARS: compensatory anti-inflammatory response syndrome, *IL*-*6*, interleukin-6
TNF-*α*: tumor nuclear factor
SAPS II: Simplified Acute Physiology Score
SOFA: Sequential Organ Failure Assessment
GCS: Glasgow Coma Scale
CLL: chronic lymphocytic leukemia
HIV: infection with the human immunodeficiency virus
HELICS: Hospital in Europe Link for Infection Control through Surveillance project
BAL: broncho-alveolar lavage
CFU: coloning-forming unit
SIRS: systemic inflammatory response syndrome
IPTW: inverse probability of treatment weighted
IL-10: interleukin-10
mHLA-DR expression: monocytes dendritic cell HLA-D expression
