# Supplementary material for: Persistent lymphopenia is a risk factor for ICU-acquired infections and for death in ICU patients with sustained hypotension at admission
Source: Ann Intensive Care. 2017 Mar 17;7:30. doi: 10.1186/s13613-017-0242-0 (PMC5355405; doi:10.1186/s13613-017-0242-0)
Supplement: Supplementary file 1 — Additional file 1. Lymphocyte_data-set. [file 13613_2017_242_MOESM1_ESM.docx]

***Electronic supplement to the article entitled:***

**Persistent lymphopenia is a risk factor of ICU-acquired infections and of death in ICU patients with sustained hypotension at admission.**

Christophe ADRIE MD, PhD^1^, Maxime LUGOSI MD^2, 3^, Leila POTTON MD^2^, Bertrand SOUWEINE MD PhD^3^, Stéphane RUCKLY MSc^6^, Jean Charles CARTIER^2^, MD, Maité GARROUSTE-ORGEAS MD PhD^4^, Carole SCHWEBEL MD PhD^2^, and Jean-François TIMSIT MD PhD^6,7^

**Table E1: Results of the model fitting the propensity score for the risk of developing an ICU-acquired infection**

| **Variables** | **Odds Ratio** | **Confidence Interval**  **95% (Wald test)** | | **Pr > Khi-2** |
| --- | --- | --- | --- | --- |
| **Age (>69 years)** | 0.924 | 0.62 | 1.37 | 0.69 |
| **Male** | 1.650 | 1.09 | 2.49 | 0.02 |
| **Admission category (reference: Scheduled surgery)** |  |  |  | 0.40 |
| Medical | 1.508 | 0.67 | 3.37 |  |
| Unscheduled surgery | 1.878 | 0.75 | 4.68 |  |
| **Centre (reference: center A)** |  |  |  | <.01 |
| B | 1.057 | 0.57 | 1.95 |  |
| C | 2.099 | 0.85 | 5.15 |  |
| D | 2.675 | 1.46 | 4.88 |  |
| **McCabe score (reference: no expected death)** |  |  |  | 0.51 |
| Death expected within 1 to 5 years | 1.053 | 0.70 | 1.59 |  |
| Death expected within a year | 0.642 | 0.28 | 1.47 |  |
| **Chronic failure (Knaus definitions)** |  |  |  |  |
| Chronic hepatic failure | 0.259 | 0.08 | 0.83 | <.01 |
| Chronic cardiovascular failure | 1.231 | 0.71 | 2.11 | 0.46 |
| Immunosuppression | 1.248 | 0.41 | 3.70 | 0.69 |
| **Long-term corticosteroids use** | 1.028 | 0.24 | 4.42 | 0.97 |
| **Chemotherapy** | 0.984 | 0.31 | 3.11 | 0.98 |
| **Complicated diabetes** | 1.021 | 0.50 | 2.07 | 0.95 |
| **Specific organ SOFA score (>2)** |  |  |  |  |
| Cardiovascular | 1.304 | 0.77 | 2.22 | 0.32 |
| Neurologic | 0.577 | 0.32 | 1.03 | 0.06 |
| Hepatic | 1.098 | 0.28 | 4.34 | 0.89 |
| Renal | 1.252 | 0.79 | 1.99 | 0.34 |
| Coagulation | 1.136 | 0.51 | 2.54 | 0.75 |
| **Gastro-protective drug** | 1.140 | 0.72 | 1.81 | 0.58 |
| **Antibiotics** | 0.639 | 0.38 | 1.06 | 0.08 |
| **Corticosteroids** | 0.910 | 0.57 | 1.45 | 0.69 |
| **Mechanical ventilation** | 1.492 | 0.82 | 2.72 | 0.19 |
| **Acute Respiratory Distress Syndrome**  **(reference : P/F>300)** |  |  |  | 0.22 |
| 200<P/F<300 | 1.159 | 0.67 | 1.99 |  |
| 100<P/F<200 | 1.663 | 1.00 | 2.75 |  |
| P/F<100 | 1.576 | 0.80 | 3.08 |  |
| **Central venous catheter** | 2.072 | 1.07 | 4.02 | 0.03 |
| **Pulmonary arterial catheter** | 1.578 | 0.62 | 3.98 | 0.33 |
| **Temperature (reference: between 36 et 39°C)** |  |  |  | 0.01 |
| Temperature>39°C | 0.885 | 0.53 | 1.48 |  |
| Temperature<36°C | 5.045 | 1.60 | 15.93 |  |
| **Mean symptom at admission** |  |  |  |  |
| Multi-organ failure | 4.097 | 1.42 | 11.78 | <.01 |
| Septic shock | 1.234 | 0.65 | 2.32 | 0.51 |
| Cardiogenic shock | 2.210 | 0.94 | 5.17 | 0.07 |
| Respiratory failure | 2.143 | 1.21 | 3.80 | <.01 |
| Coma | 2.097 | 1.05 | 4.19 | 0.04 |

AUC: 0.727; Hosmer-Lemeshow statistics: 0.6137. *P/F*: PaO_2_/FiO_2_.

SOFA: Sequential Organ Failure Assessment

**Table E2: Results of the model fitting the propensity score for the risk of ICU mortality.**

| **Variables** | **Odds Ratio** | **Confidence Interval**  **95% (Wald test)** | | **Pr > Khi-2** |
| --- | --- | --- | --- | --- |
| **Age (>69 years)** | 1.026 | 1.01 | 1.04 | <.01 |
| **Male** | 0.779 | 0.50 | 1.21 | 0.26 |
| **Admission category (reference: Scheduled surgery)** |  |  |  | <.01 |
| Medical | 0.642 | 0.21 | 1.92 |  |
| Unscheduled surgery | 2.575 | 1.08 | 6.14 |  |
| **Centre (reference: center A)** |  |  |  | <.01 |
| B | 1.076 | 0.36 | 3.25 |  |
| C | 3.993 | 1.33 | 11.97 |  |
| D | 2.038 | 0.72 | 5.76 |  |
| **McCabe score (reference: No expected death)** |  |  |  | <.01 |
| Death expected within 1 to 5 years | 5.815 | 2.65 | 12.74 |  |
| Death expected within a year  **Co-morbidities (Knaus’s definitions)** | 1.261 | 0.80 | 1.99 |  |
| Chronic hepatic failure | 2.667 | 1.16 | 6.15 | 0.02 |
| Chronic cardiovascular failure | 1.076 | 0.60 | 1.94 | 0.81 |
| Chronic respiratory failure | 0.543 | 0.31 | 0.94 | 0.03 |
| Immunosuppression | 0.409 | 0.095 | 1.755 | 0.23 |
| **Long term Corticosteroids use** | 2.337 | 0.40 | 13.59 | 0.34 |
| **Chemotherapy** | 2.184 | 0.56 | 8.47 | 0.26 |
| **Diabetes with complications** | 0.514 | 0.21 | 1.24 | 0.14 |
| **Specific organ SOFA score (>2)** |  |  |  |  |
| Cardiovascular | 1.447 | 0.78 | 2.70 | 0.25 |
| Neurologic | 1.429 | 0.81 | 2.50 | 0.21 |
| Coagulation | 1.095 | 0.46 | 2.58 | 0.83 |
| Hepatic | 1.904 | 0.59 | 6.13 | 0.28 |
| Renal | 2.121 | 1.32 | 3.40 | <.01 |
| **Corticosteroids** | 1.271 | 0.78 | 2.06 | 0.33 |
| **Mechanical ventilation** | 1.452 | 0.76 | 2.75 | 0.25 |
| **Acute Respiratory Distress Syndrome** | 1.359 | 1.10 | 1.68 | <.01 |
| **Pulmonary arterial catheter** | 1.877 | 0.69 | 5.09 | 0.21 |
| **Central venous catheter** | 2.117 | 0.91 | 4.93 | 0.08 |
| **Arterial Catheter** | 1.081 | 0.54 | 2.16 | 0.82 |
| **Temperature (reference: temperature**  **between 36 et 39°C)** |  |  |  | 0.05 |
| Temperature>39°C | 1.009 | 0.58 | 1.74 |  |
| Temperature<36°C | 4.524 | 1.23 | 16.70 |  |
| **Continuous monitoring** | 1.051 | 0.47 | 2.33 | 0.90 |
| **Cardiogenic shock** | 2.343 | 1.06 | 5.20 | 0.04 |

AUC: 0.800; Hosmer-Lemeshow statistics: 0.8327

ICU: Intensive Care Unit; SOFA: Sequential Organ Failure Assessment
